# Development of a Nationally Coordinated Evaluation Plan for the Ghana National Strategy for Key Populations

**DOI:** 10.4172/2155-6113.1000389

**Published:** 2014-12-02

**Authors:** Heidi W Reynolds, Kyeremeh Atuahene, Elizabeth Sutherland, Richard Amenyah, Isaiah Doe Kwao, Emmanuel Tettey Larbi

**Affiliations:** 1 MEASURE Evaluation, Carolina Population Center, University of North Carolina at Chapel Hill, 400 Meadowmont Village Circle, 3rd Floor, Chapel Hill, NC 27517, USA; 2 Ghana AIDS Commission, P.O. Box CT5169, Accra, Ghana; 3 Technical Assistance and Capacity Building Manager, Technical Support Facility for West and Central Africa, UNAIDS, Ouagadougou, Burkina Faso, Africa

**Keywords:** Evaluation, Ghana, Monitoring and evaluation, HIV prevention, Key populations, Data-informed decision making, Health information systems, Ownership, Government programs

## Abstract

**Objective:**

Just as HIV prevention programs need to be tailored to the local epidemic, so should evaluations be country-owned and country-led to ensure use of those results in decision making and policy. The objective of this paper is to describe the process undertaken in Ghana to develop a national evaluation plan for the Ghana national strategy for key populations.

**Methods:**

This was a participatory process that involved meetings between the Ghana AIDS Commission (GAC), other partners in Ghana working to prevent HIV among key populations, and MEASURE Evaluation. The process included three two-day, highly structured yet participatory meetings over the course of 12 months during which participants shared information about on-going and planned data and identified research questions and methods.

**Results:**

An evaluation plan was prepared to inform stakeholders about which data collection activities need to be prioritized for funding, who would implement the study, the timing of data collection, the research question the data will help answer, and the analysis methods. The plan discusses various methods that can be used including the recommendation for the study design using multiple data sources. It has an evaluation conceptual model, proposed analyses, proposed definition of independent variables, estimated costs for filling data gaps, roles and responsibilities of stakeholders to carry out the plan, and considerations for ethics, data sharing and authorship.

**Conclusion:**

The experience demonstrates that it is possible to design an evaluation responsive to national strategies and priorities with country leadership, regardless of stakeholders' experiences with evaluations. This process may be replicable elsewhere, where stakeholders want to plan and implement an evaluation of a large-scale program at the national or subnational level that is responsive to national priorities and part of a comprehensive monitoring and evaluation system.

## Introduction

A wide array of HIV prevention approaches exist, and HIV prevention programs should be tailored to local epidemic to best respond to the diverse needs of people at risk of HIV. There is international endorsement for combination (comprehensive) HIV prevention programs that include behavioural (e.g., education to encourage safer behaviours), biomedical (e.g., medical technologies such as condoms and antiretroviral prophylaxis), and structural interventions (e.g., stigma reduction) [1,2]. From this general guidance, each country needs to define a specific package of interventions that are responsive to the local epidemic, evaluate their implementation, and adjust programs and policies based on the results.

Evaluations are conducted to obtain evidence that can inform judgments about a program's performance, to improve the effectiveness of programming, for program accountability and transparency, and to inform decisions about future policies and programming including scale up [3]. Just as HIV prevention programs need to respond to the local conditions, there are increasing calls for evaluations to be country-led and aligned with country strategies [4]. Similarly, the U.S. President's Emergency Plan for AIDS Relief (PEPFAR) calls for evaluations that are driven by country needs and engagements [3]. Evaluations with high country engagement can ensure that the results or strategic information being generated are aligned with a country's own information needs, timelines, and priorities and with their stated national goals and objectives. Evidence that is aligned with what stakeholders need to know can enhance trust in the data and lead to increased data use and drive evidence based decision making and policy [5,6].

Rigorous evaluations of comprehensive HIV prevention programs have faced a number of challenges in their implementation. The pathway through which comprehensive programs influence health outcomes is complex. Because of the nature of HIV prevention programs they are targeted where the need is greatest, thereby complicating the identification of groups that serve as comparisons or control. Similarly, in countries there are a number of programs operating making it difficult to find untouched comparison groups [7].

In response to the uncertain and incomplete evidence to inform HIV prevention programs, the Joint United Nations Programme on HIV/AIDS (UNAIDS) following a call from the UNAIDS Monitoring Evaluation Reference Group (MERG) issued guidance in 2010 on evaluating HIV prevention programs [8]. The document responds to needs for practical evaluation guidelines using appropriate methods and evaluations that are unified with monitoring and evaluation (M&E) systems and grounded in the realities of the field. Central to the document are the principles of making better use of existing data for evaluation purposes and to prioritize information needs and fill data gaps. At the same time, an inter-agency group developed comprehensive guidelines to support strengthening monitoring and evaluation systems so that they are responsive to local HIV epidemics among sex workers, men who have sex with men, and transgender people [9].

MEASURE Evaluation was interested to see how this guidance informs a practical approach at the country level; an approach that puts evaluation priorities and design decisions in the hands of country decision makers, makes better use of available M&E data for evaluation purposes, and better aligns data production with information needs. Thus, in the summer of 2011 the Ghana AIDS Commission (GAC) and MEASURE Evaluation launched a process to develop an evaluation plan for an evaluation of the effectiveness the national program to prevent HIV among key populations, specifically men who have sex with men (MSM) and female sex workers (FSW), with support from the U.S. Agency for International Development (USAID)^1^.

The HIV epidemic in Ghana is heavily concentrated among key populations with high risk sexual behaviours such as female sex workers and men who have sex with men. This was based on survey evidence that key population groups faced HIV prevalence six to 12 times higher than the general population [10,11]. The recommendation to target key population groups for HIV prevention was outlined in the Ghana National Strategic Plan for HIV and AIDS 2011-2015 [12]. To operationalize the response, the GAC, with assistance from the national technical working group (TWG) on key populations developed a National Strategic Plan for Most At Risk Populations 2011-2015 [13] and an MARP Operational Plan Framework 2011-2013 to achieve the goals and objectives outlined in the national strategic plan [14]^2^.

The GAC and TWG want to conduct an evaluation so that they can understand the effectiveness of the strategic and operational plans for programs to prevent HIV among key populations in order to:

- Inform goals, objectives, and activities for the next national strategic planning cycle starting in 2016.
- Inform program implementation guidance to partners and standardized service delivery across partners.
- Provide generalizable information about effective HIV prevention program to other countries.

The purpose of this paper is to describe the process undertaken in Ghana to develop the document, the Evaluation Plan for the Ghana National Strategy for Key Populations [15] available at http://www.cpc.unc.edu/measure/publications/sr-13-75. The process undertaken in Ghana will be of interest to program planners, managers, and implementers in other countries and across health areas who want to plan and implement an evaluation responsive to national priorities, of a large scale program at the national or subnational level, and that is part of a comprehensive M&E system.

## Methods

### Overview

The process undertaken in Ghana to develop the evaluation plan was a participatory one that involved meetings between the GAC members, TWG members, and MEASURE Evaluation staff. After the GAC, MEASURE Evaluation, and USAID reached an agreement to launch the process, the methods consisted of three two-day meetings and a final meeting of the TWG to approve the evaluation plan. This occurred over the course of 12 months, from September 2011 to September 2012. After each two-day meeting, a meeting report was prepared and necessary inputs into the evaluation plan process were developed and shared with participants to review before the next meeting. The methods to developing the evaluation plan are described in more detail below.

Each of the three meetings was called by the GAC and an invitation went out to all members of the TWG from the GAC, and the GAC convened the meetings. Both the GAC and MEASURE Evaluation staff participated in developing the meeting objectives and agendas, contributing to meeting content, and facilitating the meetings. MEASURE Evaluation was responsible for producing meeting reports and documentation necessary to produce the evaluation plan. USAID funds via the MEASURE Evaluation project supported the cost of the meeting venue, per diem and transport for participants from outside Accra, and MEASURE Evaluation staff time.

Meeting participants included representatives from GAC, Ghana Health Service, and the National AIDS Control Program (NACP); donors such as USAID, U.S. Centres for Disease Control and Prevention (CDC), and German International Cooperation (GIZ); multinational organizations such as UNAIDS and United Nations Population Fund (UNFPA); local universities such as the University of Ghana School of Public Health; international nongovernmental organizations (NGOs) such as FHI 360; local government sectors such as police and prisons; and local civil society organizations providing services and support to key populations such as Centre for Population Education and Human Rights Ghana (CEPEHRG), West African Programme to Combat AIDS and STIs (WAPCAS), Prolink Organisation, and Maritime Precious Life Foundation. Meeting participants also participated in providing meeting content through both presentations and in working groups.

### Meeting 1

The first two-day meeting was held in Accra in September 2011. The purpose of the meeting was to gain an understanding of the ongoing and planned monitoring, evaluation, and research activities related to key populations going on in Ghana and to plan for continuous, coordination and effective evaluation of key populations programming. Two main documents were used to guide the content of the meeting:

- Strategic Guidance for Evaluating HIV Prevention Programs [8].
- Operational Guidelines for Monitoring and Evaluating HIV Programmes for Female Sex Workers, Men who have Sex with Men and Transgender People [9].

**Figure 1:**
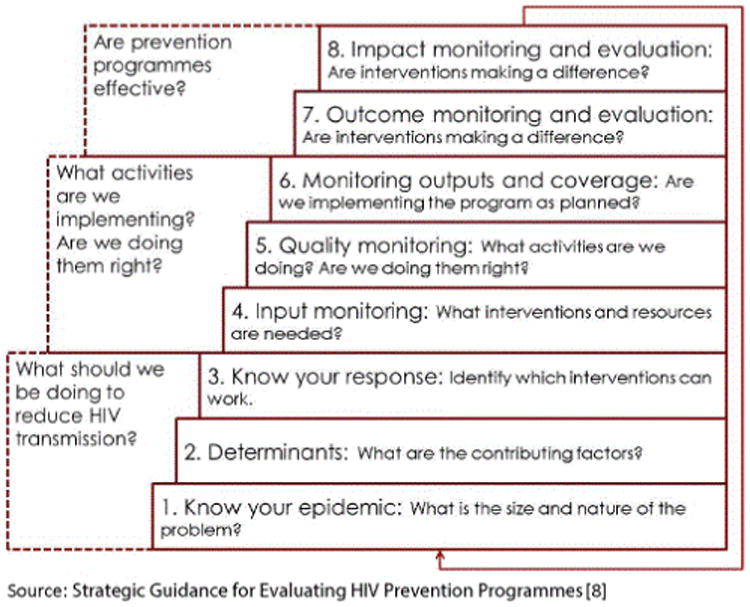
Public Health Questions Approach to HIV M&E.

Both of these documents contain the framework, “the public health questions approach to HIV M&E” (PHQA) (Figure 1). We used this framework to organize the on-going and planned M&E and research activities in Ghana. When applied at a national level, the public health questions approach to M&E should:

- Serve as a framework to organize on-going M&E activities.
- Reveal data gaps.
- Facilitate articulation of information needs relative to decision points.
- Facilitate planning to fill data gaps; and, ultimately.
- Obtain a thorough understanding of the HIV epidemic and programmatic response.

In the PHQA, each of the eight components needs to be addressed in order to have a comprehensive M&E system, and each step is aligned with related research questions and data collection needs.

The activities carried out in this first meeting included presentations by participants on their various researches and monitoring and evaluation activities on-going in Ghana in order to take stock of the current and planned research and M&E activities. The details about the M&E and research activities presented during the meeting were mapped to the steps in the PHQA.

Other activities at this first meeting included presentations on the UNAIDS approach to evaluating HIV programs [8], a general overview of different evaluation designs, and the idea for developing and evaluation plan for Ghana. From the information provided at the meeting and from existing documentation, we also constructed a logic model for the national key populations program. Logic model are commonly used in program evaluation and help visualize the relationships among resources, activities, and results [17]. Use of a logic model in combination with the PHQA framework is useful for identifying gaps in M&E activities and to prioritize how to fill those gaps. To fill the activities and outputs in the logic model, we drew from the key population operational plan framework [14]. The logic model outcomes were drawn from the national strategic plan and key populations strategy [12,13]. The impact is from the national strategic plan (reduction of new HIV infections by 50% in 2015) [12].

Using existing documents to construct a logic model for the national HIV prevention program went a long way. However, the model was incomplete particularly in terms of what were considered the key program outcomes. Also lacking was an articulated program theory of change. Thus, we formed small groups to provide inputs on the theoretical program impact pathways and the main process, output and outcome measure from the logic model. We also had discussed the indicators and data sources for those measures.

### Meeting 2

A second meeting was held in March 2012 in Accra. At this meeting, the objective was to obtain details necessary to draft the evaluation plan for the national key populations program, including identifying research questions; identifying the appropriate and feasible study designs; verifying on-going M&E activities; and discussing the main program impact pathways and gaps in data, including defining program “reach”.

One of the first steps to designing an evaluation is to identify the research questions [18]. At the meeting, presenters provided overviews of types of research questions and the strength of evidence produced by different study designs. The point was to demonstrate how the selection of a research question should be based on what is necessary to know and for what purpose, not based on what is nice to know. The research question informs the appropriate study design and data collection methods. The appropriate study design will yield a certain “level of evidence” that, in turn, influences how certain one is in the results and how the results can be interpreted to inform policy and program recommendations. In this second meeting, participants in working groups were asked to identify “What evidence will represent success of the key populations program in 2015?” The objective was to get participants to think about what answers they want in 2015 about their program to prevent HIV infections among key population groups.

With deeper knowledge about the on-going and planned M&E and research data, it became clearer that a distinction between plausibility and probability study designs was needed. Plausibility designs are appropriate when random assignment into intervention and control groups is not feasible and when the program being evaluated includes interventions of known effectiveness [19]. In Ghana, programs have been targeted to areas with greatest need, and answers are needed more for learning and to inform future strategic plans than to understand the probability that an intervention caused a particular change. Plausibility evaluations do have control or comparison groups, but they may be non-random or employ other types of controls such as historical control groups or control groups constructed based on program exposure [19]. It is important to document potential confounders when undertaking plausibility evaluations to try to control for other alternative explanations of program effect on outcomes. Moreover, information about program implementation is important for plausibility evaluations since this information is important to understand how programs are being implemented, particularly in order to help explain “why” changes in outcomes are observed. Probability evaluations require a randomly assigned control or comparison area as alternative explanations are ruled out largely under the assumptions associated with random assignment [19].

Participants also discussed the definitions of the main independent variables, particularly program “reach”. The goal of the national key populations' strategy is to reach 80% of key population at risk of HIV by 2015. To be able to measure whether the goal was accomplished, an operational definition of “reach” is needed at the national level to know whether a person was reached by the program. The concept of “program reach” is comprised of different types of service contacts [9]. An example of a service contact is when an individual gets an HIV test, receives counselling on safe sex, is screened for sexually transmitted infections, etc. To define program reach, we consulted several documents including the National HIV and AIDS Monitoring and Evaluation Plan 2011-2015 [12]; the Operational Guidelines for Monitoring and Evaluation of HIV Programmes for Sex Workers, Men who have Sex with Men, and Transgender People [9]; and the United Nations General Assembly Special Session (UNGASS) on HIV/AIDS' relevant guidelines and definitions [20]. At the meeting, participants worked to define an operational definition of “reach” that includes priority interventions, defines what is considered a contact by an individual with the services, defines the number of contacts in a period of time, is able to be measured by existing data collection forms and tools (or can be developed), and avoids double counting.

### Meeting 3

In between the second and third meetings, the Evaluation Plan for the Ghana National Strategy for Key Populations was drafted. The objective of that final two-day meeting in June 2012 was to obtain TWG member input and details necessary to finalize the draft evaluation plan for the national program to prevent HIV infections among key populations. Discussion points included:

- Feedback related to the study rationale, design and analysis plan.
- Input into priority study measures and
- Roles and responsibilities for action items for carrying out proposed data collection activities including identifying sources of funding for data gaps and responsibly parties to follow up on action steps.

Information obtained during the third meeting was used to revise and finalize the evaluation plan, which was submitted to the TWG in September 2012 and presented at the Ghana National HIV and AIDS Research Conference in September 2013. The evaluation plan was subsequently posted to MEASURE Evaluation's Web site and a onetime printing of 800 copies was made for stakeholders in Ghana.

## Results

The main outputs from the meetings included the following:

- Planned and on-going M&E and research activities mapped to the eight steps of the PHQA framework and the anticipated result of those activities was an important output. For example, for Step 1, “Know your epidemic: What is the size and nature of the problem” the on-going M&E activity was an integrated biological and behavioural surveillance survey (IBBSS) with MSM and FSW. Anticipated results from that activity were size estimates, denominator for coverage estimates, HIV prevalence estimates, and behavioural data.
- Information needs and data gaps were identified. For example, participants identified the need to better understand the role of substance abuse among MSM and risk behaviours. This information could be obtained through formative and qualitative studies. The identified gaps and information needs were relatively exhaustive, and although not all will be used in the evaluation plan, they are now documented and may be useful for other efforts.
- A logic model of defined activities, outputs, outcomes and impact for the national key population program was produced.
- Primary and secondary research questions were defined and prioritized by participants.
- Research methods and date sources needed to be able to execute the study design and carry out data analyses to answer the research questions were identified.
- Information was gathered about main outcome variables and data sources to help answer the research questions.
- Preliminary definitions of the main independent variables, program “reach”, and “exposure” were identified.
- A timeline for implementing data collection and analysis in order to yield results in time for the next strategic planning process was developed.

The research questions prioritized by participants for the evaluation plan were as follows:

- Are changes in outcomes (or changes in HIV prevalence, incidence or STI prevalence over time) due to the implementation of services and program components?
- Are there changes in behavioural outcomes and HIV prevalence and incidence over time?
- To what extent are planned MSM and FSW program activities realized/implemented and with improved quality?

To be able to answer these questions, data from a planned survey in 2015 will help answer whether being reached by the program is associated with outcomes compared with those not reached by the program. Survey data need to be complemented by contextual data to reveal possible factors that may have modified the relationship between program reach and outcomes. Data from the surveys at two time points (one in 2011 and one planned for 2015) are necessary to understand changes in outcomes over time. Analysis of existing routine program data will help understand trends in program implementation and coverage in terms of condom promotion, HIV testing, etc. Data from process evaluations will help clarify the extent to which programs are being implemented as planned and whether quality improves over time. Comparing results from the different data sources can help show whether changes in outcomes were occurring at the same time that program reach was intensifying. Based on the study design, however, it will not be possible to say with certainty that the program resulted (or “caused”) these changes.

**Figure 2:**
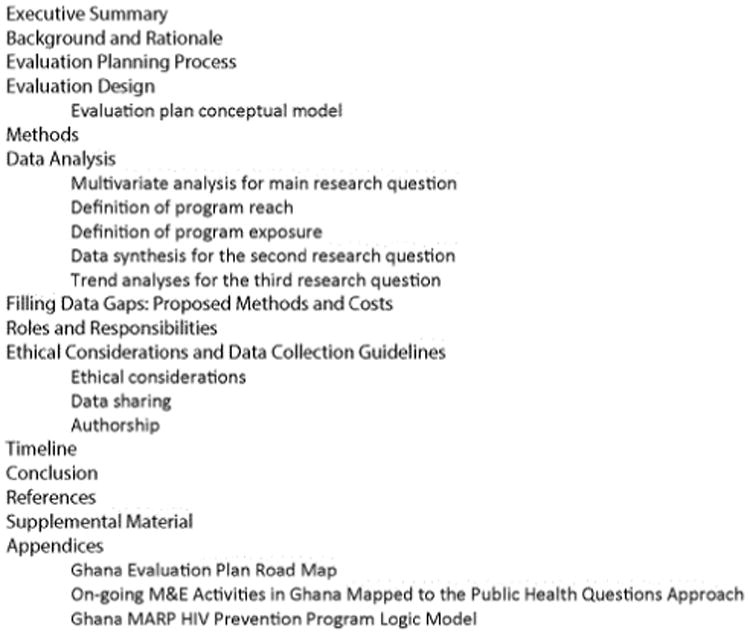
Ghana Evaluation Plan Contents [15].

The ultimate result from these meetings was an evaluation plan that serves as a roadmap for collecting important data for programmers which helps in identifying gaps, improving quality and scaling up the programs without diluting quality of programs. The plan informs stakeholders about which data collection activities need to be prioritized for funding, whose role it is to implement the study, the timing of data collection, the research question the data will help answer, and the analysis methods (figure 2). The evaluation plan includes a discussion of various methods that can be used, including the recommendation for the plausibility design using multiple data sources. In addition, it provides a method for measuring changes of various indicators over time. The plan has an evaluation conceptual model; proposed multivariate analyses; proposed definition of independent variables; estimated costs for filling data gaps; roles and responsibilities of stakeholders to carry out the plan; and considerations for ethics, data sharing, and authorship.

Overall, the evaluation plan has given added value to the work of the Ghana AIDS Commission in ways that were unanticipated originally. The plan has been catalytic in galvanizing support for key population related surveys and evaluation especially, in the area of resource mobilization. As a result, partners have made resources available for population size estimation and a planned 2015 IBBSS among MSM, FSW and their clients, and people who inject drugs. These studies will measure trends in the HIV epidemic and other sexually transmitted infections and track the outcomes of the implementation of the key population national strategy, a key objective of the evaluation plan. The evaluation plan has strengthened the GAC's efforts at results-based management practices where rigorous analysis and evidence-based approaches ensure program effectiveness and better health outcomes.

## Discussion

This paper presents the experience and process in Ghana of designing an evaluation plan. The experience demonstrates that with stakeholder agreement, funding, and parties committed to carrying out the process, it is possible to design an evaluation plan that is responsive to national strategies and priorities. Although this process relied on some donor funding and external facilitation, this process respected the GAC's leadership and facilitated their ability to exercise their leadership. The process is informed by international recommendations [8,9] and reported here in such a way that it could be replicated or adapted in other settings.

There were a number of factors that facilitated our ability to launch this activity in Ghana. There was strong national leadership, a clear national strategic plan and goals, and an active national level TWG composed of government, donors, and program implementers. The timing of developing the evaluation plan coincided with the GAC's desire to build evidence of the effectiveness of the strategic and operational plans to inform future strategic plans, to develop guidance to partners implementing programs, and to contribute to the global evidence base about what works for HIV prevention programs for key populations. Although there were no obvious control groups, there were on-going population based surveys that could serve as baselines.

We hypothesize that this process may be applicable elsewhere, but will likely be successful in a context with facilitating characteristics such as strong national leadership and national strategic plans that have legitimacy with and buy-in from other stakeholders. Although the timeframe and workshop agendas were highly specific to the Ghana context, resources cited in this document, particularly the UNAIDS guidelines and the PHQA framework, are publically available to help tailor the process to other settings.

The main limitation is that the process to develop the evaluation plan has not been demonstrated to be fully successful; only when the plan is used to carry out an actual evaluation will it be a success. It is important to note, however, that as a result of the evaluation planning processes, one activity has already been prioritized and funded, a performance evaluation of the national key populations program [21]. Confirmation of other investments in data collection activities are needed. Thus, the disadvantage of this approach is that the evaluation could be endangered (and resources wasted) if sufficient high quality data are not collected and analysed as planned. Strategies to raise the likelihood that evaluation plans are implemented include ensuring strong country leadership and wide partner support for the process; implementing the process that corresponds with budget planning; and specifying resources needed and source of funds.

Other potential limitations include the time and the cost. The evaluation plan was created over the course of one year. The time frame was dependent on stakeholder's availability, as it is hard to find times for national level stakeholders to free themselves up for two full working days given their other responsibilities. On the other hand, the evaluation planning period occurred early in the strategic planning cycle, allowing for sufficient time for needed data to be collected and analysed.

The approach incurred financial costs for per diems and venue rentals, for example, as well as opportunity costs. When people attended the meetings, this took them away from their other job responsibilities. Covering the financial costs can be planned for in annual budgets, and the activity of planning and conducting and evaluation can be integrated into stakeholders' work plan cycles. This has to be planned for in advance, however.

The experience in Ghana demonstrates an approach to planning evaluations at the national level that includes a program theory or logic model, links decisions that need to be made to planning for data collection, and uses existing data sources where possible. To our knowledge this was the first time that all parties working on key populations programs and M&E and research in Ghana had a chance to meet and discuss the full picture of activities taking place. It is possible to have a process to evaluate HIV prevention programs that is participatory, regardless of stakeholders' level and experience with evaluation.

## Footnotes

1 The national key populations strategic framework also addresses prisoners and people who use injecting drugs, but we focused on the MSM and FSW populations since these programs are most widespread.

2 In October 2011, UNAIDS issued guidance indicating that “key populations at higher risk” or just “key populations” is the preferred term over MARP or “most-at-risk populations”, since the MARP term can be stigmatizing.[16] These documents were completed prior to those recommendations and thus their titles reflect the older terminology, otherwise the preferred terminology is used wherever possible.
